# ﻿*Niphargus* Schiödte, 1849 (Crustacea, Amphipoda, Niphargidae) is a new component of the biotic community in the deep pebble beach habitats of the northern Black Sea region

**DOI:** 10.3897/zookeys.1254.165665

**Published:** 2025-10-03

**Authors:** Ivan N. Marin, Dmitry M. Palatov

**Affiliations:** 1 A.N. Severtsov Institute of Ecology and Evolution of RAS, Moscow, 119071, Russia A.N. Severtsov Institute of Ecology and Evolution of RAS Moscow Russia

**Keywords:** Barcoding, Black Sea, Caucasus, coastal habitat, diversity, phylogeography, taxonomy

## Abstract

A new species of the genus *Niphargus* Schiödte, 1849 (Crustacea: Amphipoda: Niphargidae) is described from the deep pebble beach interstitial habitats along the northern Black Sea coastline, revealing a new type of environment for this amphipod genus and providing new insight into the diversity of this unusual biotope. *Niphargus primoricus***sp. nov.** belongs to “*stygius*–*longicaudatus*” species group corresponds to a distinct phylogenetic lineage, recently called “*tarkhankuticus*” ingroup (clade), which currently includes several species from the coastal areas of the Black Sea (Crimean Peninsula, southern Caucasus, and northern coast of the Republic of Türkiye [Turkey]). Molecular genetic analysis revealed that the speciation within this ingroup started in the Pliocene, approximately 5.76–3.6 Mya, and correlated with the Black Sea transgression. The divergence of the “*tarkhankuticus*” clade from the related European congeners probably occurred in the Late Miocene (~ 11–10 Mya), and is likely related to the separation of the Paratethys into different basins (Euxinian, Alpine and Pannonian). The new species has a wide distribution, currently inhabiting nearly 190 kilometers along the Black Sea coastline, from Gelendzhik to Khosta, and is characterized by a low level of genetic divergence between populations. The deep pebble interstitial coastal biotopes in the area are also inhabited by specific gammarid amphipods, for example, *Dursogammarus dromaderus* Marin & Palatov, 2022 and *Litorogammarus dursi* Marin, Palatov & Copilaş-Ciocianu, 2023 (Amphipoda: Gammaridae), whose biology has not been studied, and it is unknown how they spread along the coastline.

## ﻿Introduction

The Caucasus is a well-known biodiversity hotspot, characterized by a unique and diverse fauna, including many stygobiotic species. Such diversity is supported and shaped by a stable climate and the separation of ecological niches and microhabitats during the recent time (Myers et al. 2000; Krever et al. 2001). The Colchis, or the Colchis Lowland of the Black Sea, a local refugium formed in the late Miocene (ca 7–6 Mya) and not exposed to glaciers, had sheltered relic organisms during the Quaternary Ice Age (ca 2.59 Mya–present) and the Last Glacial Maximum (ca 26,500 years ago), which dramatically changed the Northern Hemisphere biota (Hewitt 2000; Cane et al. 2006; Schmitt 2007; Shatilova et al. 2011; Keppel et al. 2012). In addition, the Caucasus region follows the Balkan Peninsula in terms of its karst terrain (Myers et al. 2000; Krever et al. 2001), showing a high diversity of subterranean and stygobiotic fauna similar to that of the Balkans and southern Europe (e.g., Myers et al. 2000; Krever et al. 2001; Chertoprud et al. 2016, 2021; Marin 2017; Bardjadze et al. 2018).

The genus *Niphargus* Schiödte, 1849 (Crustacea: Amphipoda: Niphargidae) is the largest freshwater amphipod genus, comprising more than 400 species (Horton et al. 2024), which inhabit a wide range of subterranean and epigean aquatic habitats, from deep cave lakes and small pores in the epikarst to helocrene spring, wells and hyporheic zone of rivers in the West Palearctic (Väinölä et al. 2008; Fišer 2012; Fišer et al. 2014). Numerous species of the genus *Niphargus* have been described from the Caucasus, but it is obvious that the fauna of the region has been studied fragmentary and its real diversity is still far from being fully known (e.g., Karaman 2012; Marin 2019; Marin et al. 2021a, b, 2023a; Marin and Palatov 2023, 2024, 2025).

The underground (stygobiotic) lifestyle appears to be an ancestral trait for the genus *Niphargus* (McInerney et al. 2014; Esmaeili-Rineh et al. 2015; Delić et al. 2016); recent species are found living in various stygobiotic environments. Recent molecular genetic studies suggest that most *Niphargus* species have a limited distribution, being mostly narrow endemics (Fišer et al. 2008, 2018, 2019; Delić et al. 2017; Eme et al. 2017), while currently known widespread stygobiotic taxa obviously represent complexes of cryptic species (Lefébure et al. 2006, 2007; Delić et al. 2017). Besides, similar to most of subterranean/stygobiotic animals, the representatives of the genus *Niphargus* are unable to live outside of their habitats, being well adapted to ecologically narrow stygobiotic/subterranean conditions (McKinney 1997; Culver and Pipan 2009; McInerney et al. 2014). They are very sensitive to environmental changes and unable to disperse over long distances due to the stenobiotic (narrow ecological niche) nature of their species (Fišer et al. 2006; Foulquier et al. 2008; Trontelj et al. 2009; McInerney et al. 2014).

Nevertheless, there are several species within the genus, for example, *Niphargus hrabei* Karaman, 1932, *Niphargus valachicus* Dobreanu & Manolache, 1933 and *Niphargus potamophilus* Birštein, 1954, as well as to some extent Caucasian *Niphargus bzhidik* Marin, Krylenko & Palatov, 2021 and the members of the “*Niphargus magnus*” species complex (Copilaş-Ciocianu et al. 2014, 2017, 2018; Marin et al. 2021a, b; Palatov and Marin 2021), the distribution of which differs from the general rule, since, being epigean, these species live in open water habitats, such as small rivers, ponds and even temporary forest puddles. However, ranges of the species distribution more than 200 km are considered the exception rather than the rule in niphargids (Trontelj et al. 2009). The question of how these species arrive at their current locations remains unanswered, but it is believed that epigean species disperse as a result of flooding or seasonal river overflow (passive processes of long-distance dispersal) (van Leeuwen et al. 2013; Copilaş-Ciocianu et al. 2018).

During the study of the deep layers of pebble beaches of the northern part of the Black Sea, where gammarids, *Dursogammarus dromaderus* Marin & Palatov, 2022 and *Litorogammarus dursi* Marin, Palatov & Copilaş-Ciocianu, 2023 (Amphipoda: Gammaridae), strictly specific to this biotope (native pebble-dwelling species) (see Marin and Palatov 2022; Marin et al. 2023a) were previously found, representatives of the genus *Niphargus* were also recovered. Similar to gammarids, this species inhabits freshwater springs located deep in pebbles layers of coastal beaches, and has a typical stygomorphic morphology, and presumably it should belong to species with a narrow range, characteristic of most species of the genus *Niphargus*. This species is described below as new to science.

## ﻿Materials and methods

### ﻿Specimen sampling

Amphipods were collected in wells and springs along the northern Black Sea coastline using hand net. Specifically, we dug out the top layer of beach pebbles by hand and a shovel in an area where small streams or rivers flowed into the sea, and then used a hand net to catch the crustaceans in the resulting water-filled cavity. After sampling, all specimens were fixed in 96% solution of ethanol for molecular-genetic study (see Table 1).

**Table 1.** The list of stations where representatives of the new *Niphargus* species were taken for molecular-genetic study (COI mtDNA gene marker).

№ of station	Coordinates	Data	№ of specimens (GenBank numbers)
**1**	44°34'35.9"N, 37°58'48.3"E	12.07.2023	PX233139, PX233140
**2**	44°22'16.67"N, 38°23'48.84"E	12.06.2024	PX233141
**3**	44°14'52.24"N, 38°50'35.37"E	08.06.2024	PX233142, PX233143
**4**	44°11'28.12"N, 38°53'05.52"E	08.06.2024	PX233144
**5**	44°08'06.75"N, 39°01'33.5"E	07.06.2024	PX233145
**6**	44°04'00.4"N, 39°08'22.8"E	13.05.2019	PX233146
**7**	43°59'34.11"N, 39°12'37.98"E	06.06.2024	PX233147, PX233148
**8**	43°57'10.72"N, 39°18'43.1"E	05.06.2024	PX233149
**9**	43°53'57.35"N, 39°20'03.97"E	03.06.2024	PX233150, PX233151
**1**0	43°30'33.9"N, 39°52'07.0"E	12.06.2024	Paratype ♀, ZMMU Mb-1300 (PX233133) (see below) + PX233134–PX233138
	43°30'55.3"N 39°52'10.1"E (type locality for the new species)	23.10.2024	Holotype ♂, ZMMU Mb-1299 (PX233131) (see below) + PX233132

The type material was deposited in the collection of Zoological Museum of Moscow State University, Moscow (**ZMMU**); additional materials were deposited in the author’s private collection deposited at the A.N. Severtsov Institute of Ecology and Evolution of RAS, Moscow (**LEMMI**).

### ﻿Morphological studies

All collected specimens were preliminarily processed, sorted based on specific morphological features. The fixed samples were dissected using a Lomo MBS-10 light binocular microscope, tweezers, and dissecting needles. The prepared limbs were placed on slides in glycerin or polyvinyl lactophenol (PVL), covered with a cover glass and bordered with transparent nail polish. Then they were photographed under an Olympus SX10 light microscope at standard magnifications of ×5, ×7, and ×10.

Scanning electron microscope (SEM) micrographs were made with standard methods using a CamScan S2 microscope in the Electronic Microscopy Laboratory of the Biological Faculty of the Moscow State University. The samples placed in 95% ethanol were cleaned in an ultrasonic cleaner, followed by dehydration with acetone and critical-point drying (CPD). Subsequently, they were affixed to specimen stubs with double-sided tape, and finally coated with gold through sputtering using the Polaron PS 100. The body length (bl., in mm) – the dorsal length from distal margin of head to the posterior margin of telson, without the length of uropod III and antennas – was used as a standard measurement.

### ﻿Molecular and phylogenetic study

A fragment of cytochrome c oxidase subunit I (COI mtDNA) (DNA barcoding) is used as one of the better-known tools for studying the interspecific and intraspecific (population) structures, species delimitations, cryptic diversity, and phylogenetic relationships (Hebert et al. 2003; Rebijith et al. 2013; Marin et al. 2021a). The samples for molecular-genetic analysis, a small piece of muscle tissue, were extracted from the abdominal segments or and pereopods of the specimens, which were then deposited in collection as vouchers. To understand the intraspecific (population) genetic structure of the new species, the holotype ♂, ZMMU Mb-1299, paratype ♀, ZMMU Mb-1300 from the Khosta River (the type locality) and individuals from sampling stations were used for the molecular-genetic analysis (see Table 1). Total genomic DNA was extracted using the innuPREP DNA Micro Kit (Analitik Jena, Jena, Germany) following the manufacturer’s protocol. The gene marker was amplified with the help of the universal primers LCO1490 (5’–GGTCAACAAATCATAAAGATATTGG–3’) and HC02198 (5’–TAAACTTCAGGGTGACCAAAAAATCA–3’) (Folmer et al. 1993) using a T100 thermocycler (Bio-Rad, Hercules, CA, USA) under the standard protocol conditions. Obtained sequences, 646 bp in length, were aligned using MEGA 7.0 (Kumar et al. 2016). The best evolutionary substitution model was determined using MEGA 7.0 and jModeltest2.1.141 (Diego Darriba, Universidade da Coruña as part of the Computer Architecture Group (GAC), Coruña, Spain) on XSEDE via the CIPRES (Cyber Infrastructure for Phylogenetic Research) Science Gateway v. 3.3 (http://www.phylo.org/). A phylogenetic analysis was conducted using PhyML 3.0 (http://www.atgc-montpellier.fr/phyml/) (Guindon et al. 2010) with several models based on BIC (Bayesian Information Criterion) and AIC (Akaike Information Criterion). This phylogenetic analysis was used to search for related species and is not visualized in the article.

**Figure 1.**
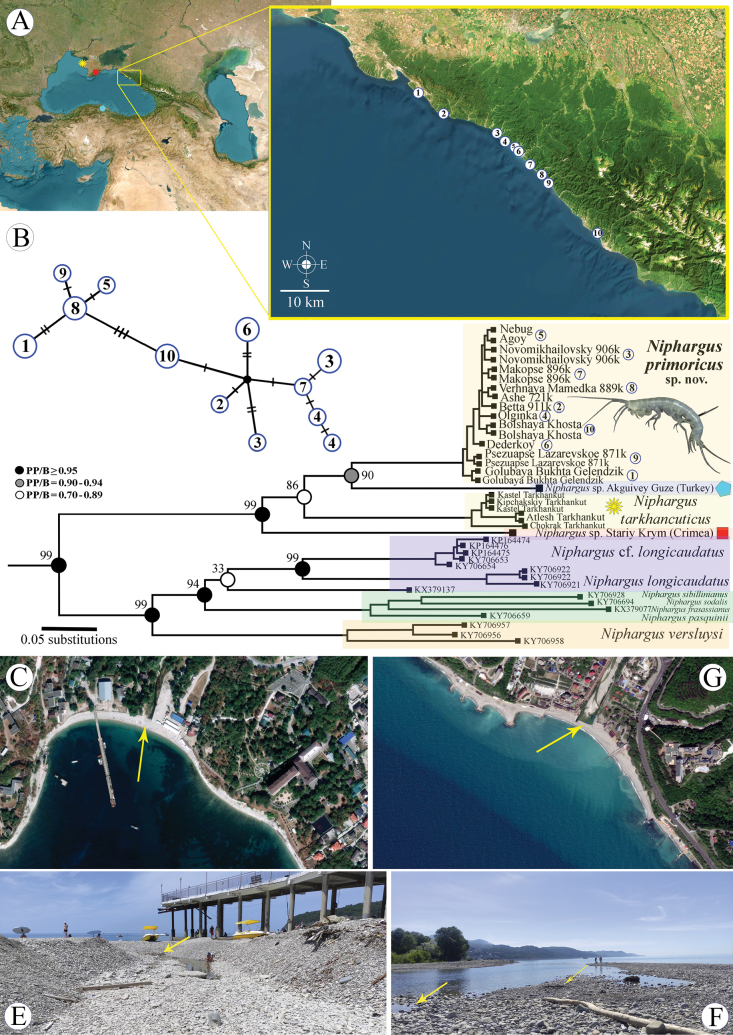
A. The map of collection sites (see Table 1); B. Schematic haplotype network and phylogenetic tree (reconstruction) of relationships of the *Niphargus primoricus* sp. nov. Characteristic biotopes, where the new species was found. C. The bay at the mouth of the Ashamba River, Golubaya Bukhta, Gelendzhik, D. The mouth of the Nebug River, Nebug, E. Peeble beach at the mouth of the Tu River, Olginka; F. Layers of pebbles at the mouth of the Psezuapse River, Lazarevskoe. Yellow arrows indicated the exact sampling localities.

The final aligned dataset for the ingroup analysis included 54 COI sequences, displaying 244 variable (polymorphic) sites, of which 234 were parsimony-informative. The dataset included both new sequences (see below) and sequences of the related European *Niphargus* species (*N. tarkhankuticus*, *N. longicaudus*, *N. subillinianus*, *N. sodalis*, *N. frasassianus*, *N. pasquinii* and *N. versluysi*) taken from the GenBank (NCBI) database (see Fig. 1B; Marin et al. 2022). Phylogenetic tree topologies were congruent between Bayesian (BL) and Maximum Likelihood (ML) analyses.

The genetic divergences (*p*-distances) were calculated with MEGA 7.0 (University of Kent, NZ) using the Kimura 2-Parameter (K2P) model of evolution (Kimura 1980).

A median joining network of haplotypes (Bandelt et al. 1999) was reconstructed using the dataset of the sequences belonging to the new species with PopArt (Population Analysis with Reticulate Trees) software (Leigh and Bryant 2015). The final dataset of the sequences of the COI mtDNA gene marker for the ingroup analysis included 17 sequences, displaying 23 variable (polymorphic) sites, of which 19 were parsimony-informative.

### ﻿Time calibration

The estimated minimum (5.16%/Mya^-1^) and maximum (as 0.77%/Mya^-1^) divergence times were calculated after Guy-Haim et al. (2018), with an average divergence time of 2.5% Mya^-1^ for COI mtDNA gene marker (Lefébure et al. 2006; Copilaş-Ciocianu and Petrusek 2015; Guy-Haim et al. 2018). Additionally, a divergence time estimates as 1.773% Mya^–1^ was calculated according to Copilaş-Ciocianu et al. (2019).

## ﻿Taxonomic account

### ﻿Phylum Arthropoda von Siebold, 1848

**Class Malacostraca Latreille, 1802**

**Order Amphipoda Latreille, 1816**

**Family Niphargidae Bousfield, 1977**

**Genus *Niphargus* Schiödte, 1849**

#### ﻿ Niphargus primoricus sp. nov.

Taxon classificationAnimaliaAmphipodaNiphargidae

8390EBB5-7754-50FD-A2DF-50FE8D95EBA5

https://zoobank.org/3ED10492-D4D5-420A-A2EF-9511C8E17C2C

**Figure 2.**
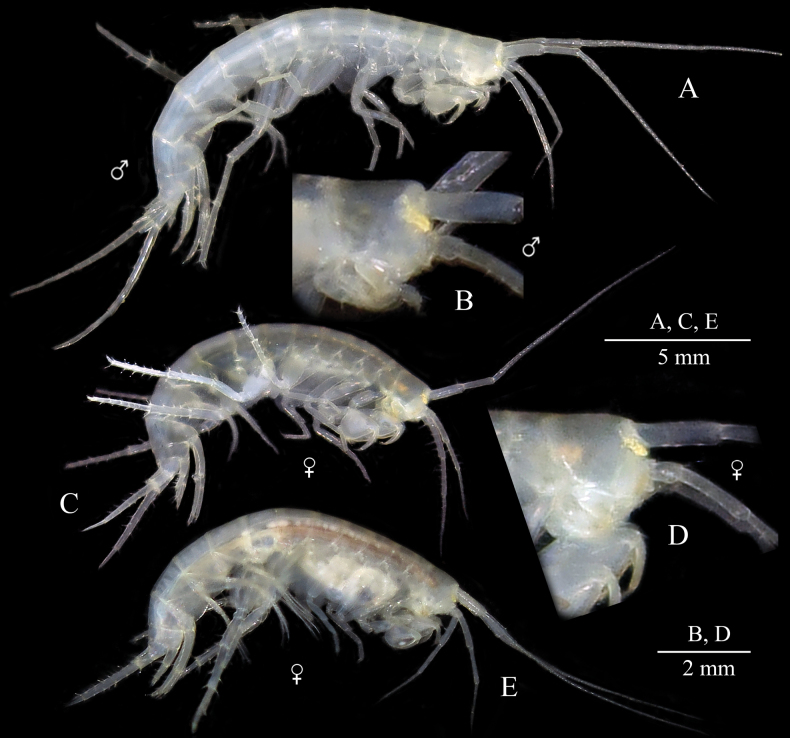
*Niphargus primoricus* sp. nov., holotype ♂ (ZMMU Mb-1299) (A, B), paratype ♀ (ZMMU Mb-1300) (C, D), and ♀ (LEMMI) (E) from the hyporhean zone of the Khosta River, Khosta. A, C, E. General lateral view; B, D. Head, enlarged.

**Figure 3.**
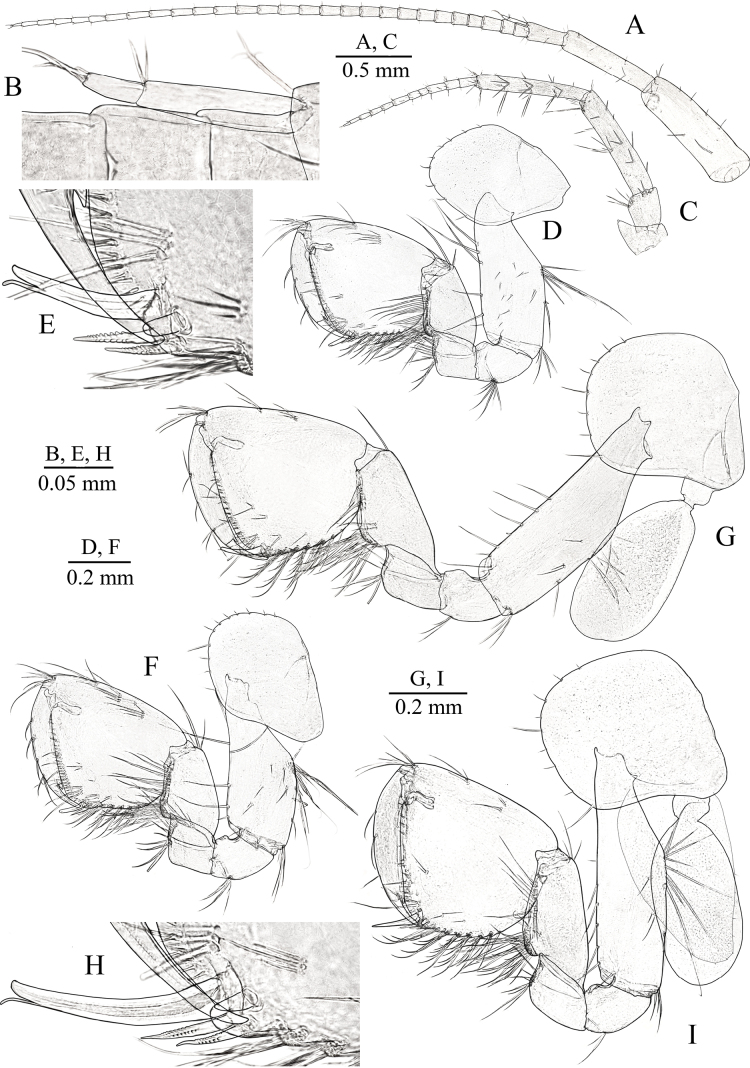
*Niphargus primoricus* sp. nov., holotype ♂ (ZMMU Mb-1299) (A–E, G, H) and paratype ♀ (ZMMU Mb-1300) (F, I). A. Antenna I; B. Accessory flagellum of antenna I; C. Antenna II; D. Gnathopod I; E. Distoventral palmar corner of propodus of GnI; F. Gnathopod I; G. Gnathopod II; H. Distoventral palmar corner of propodus of GnII; I. Gnathopod II.

**Figure 4.**
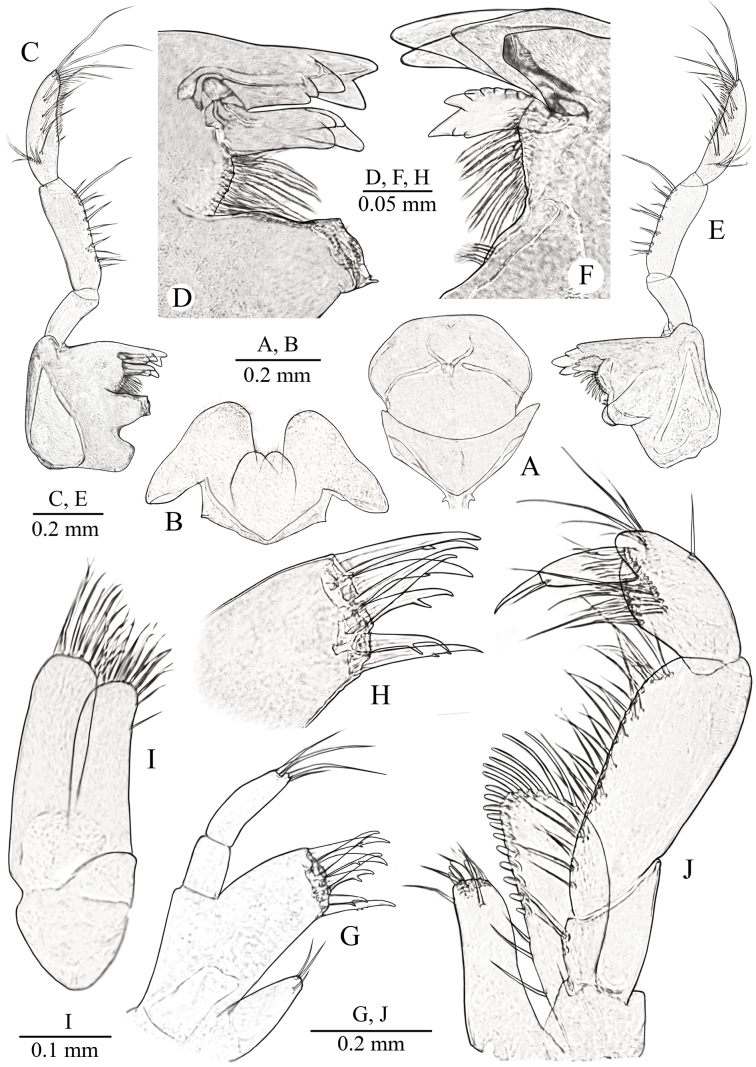
*Niphargus primoricus* sp. nov., holotype ♂ (ZMMU Mb-1299). A. Labrum (upper lip); B. Labium (lower lip); C, E. Mandible; D, F. Incisor process and pars incisiva of mandibles; G. Maxilla I; H. Same, distal margin of outer lobe; I. Maxilla II; J. Maxilliped.

**Figure 5.**
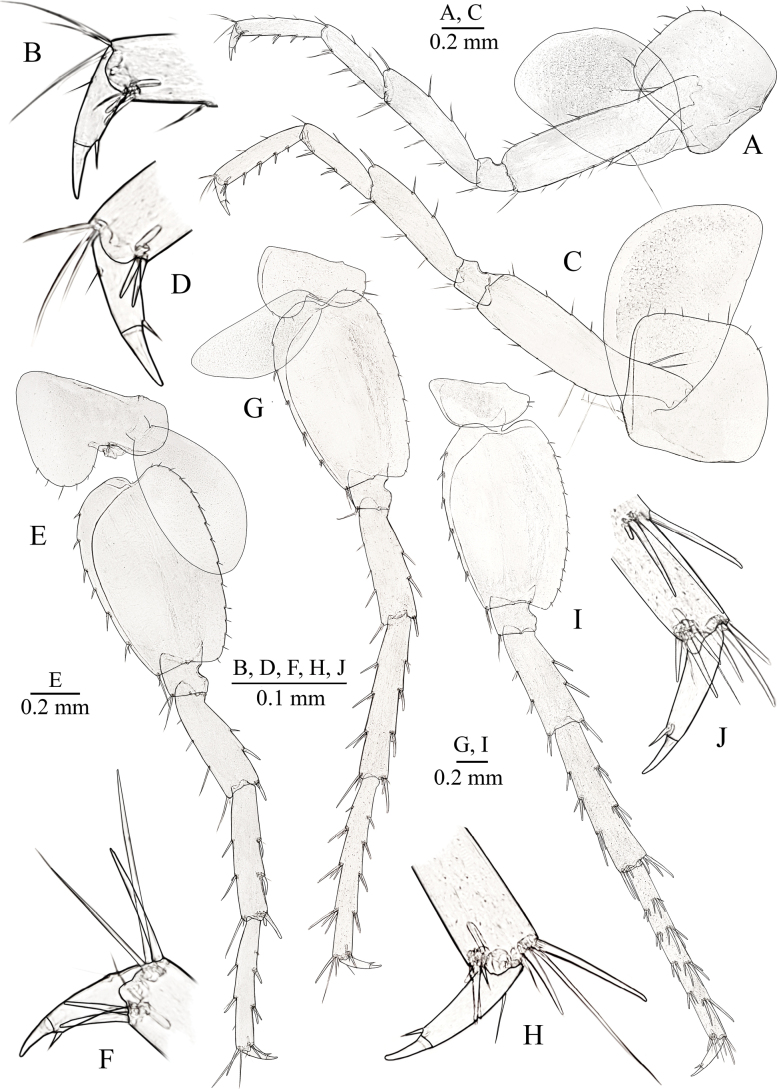
*Niphargus primoricus* sp. nov., holotype ♂ (ZMMU Mb-1299). A. Pereopod III; B. Dactylus of PIII; C. Pereopod IV; D. Dactylus of PIV; E. Pereopod V; F. Dactylus of PV; G. Pereopod VI; H. Dactylus of PVI; I. Pereopod VII; J. Dactylus of PVII.

**Figure 6.**
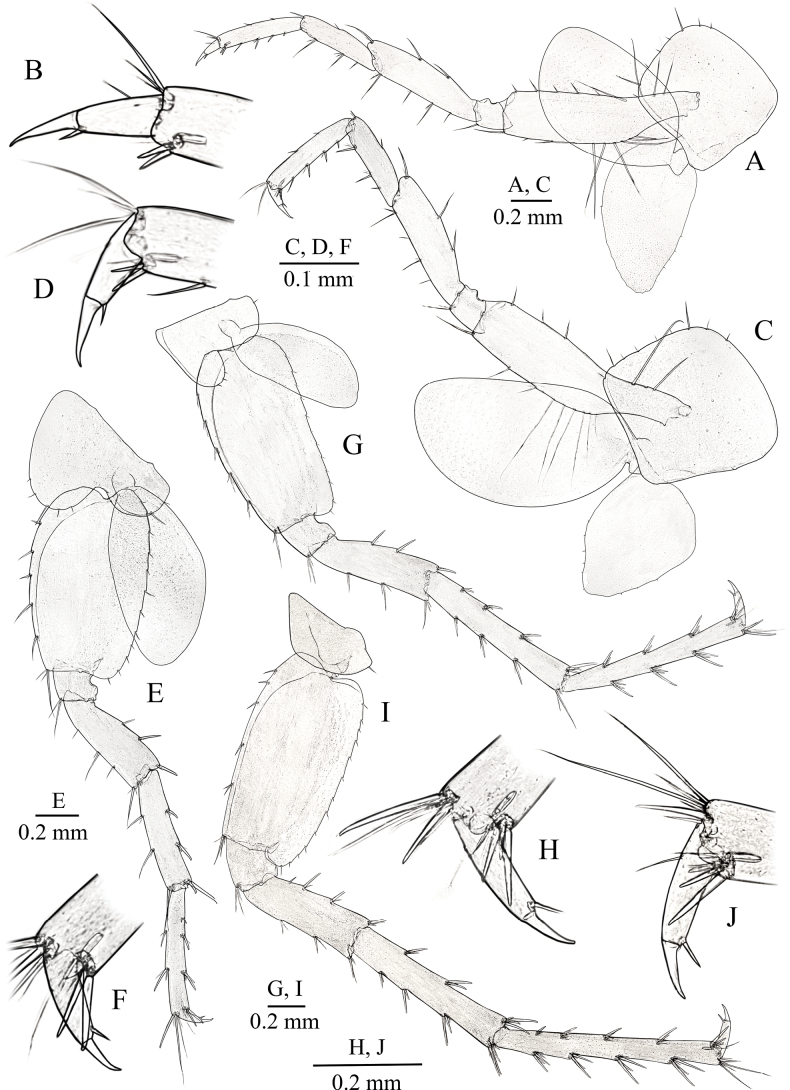
*Niphargus primoricus* sp. nov., paratype ♀ (ZMMU Mb-1300). A. Pereopod III; B. Dactylus of PIII; C. Pereopod IV; D. Dactylus of PIV; E. Pereopod V; F. Dactylus of PV; G. Pereopod VI; H. Dactylus of PVI; I. Pereopod VII; J. Dactylus of PVII.

**Figure 7.**
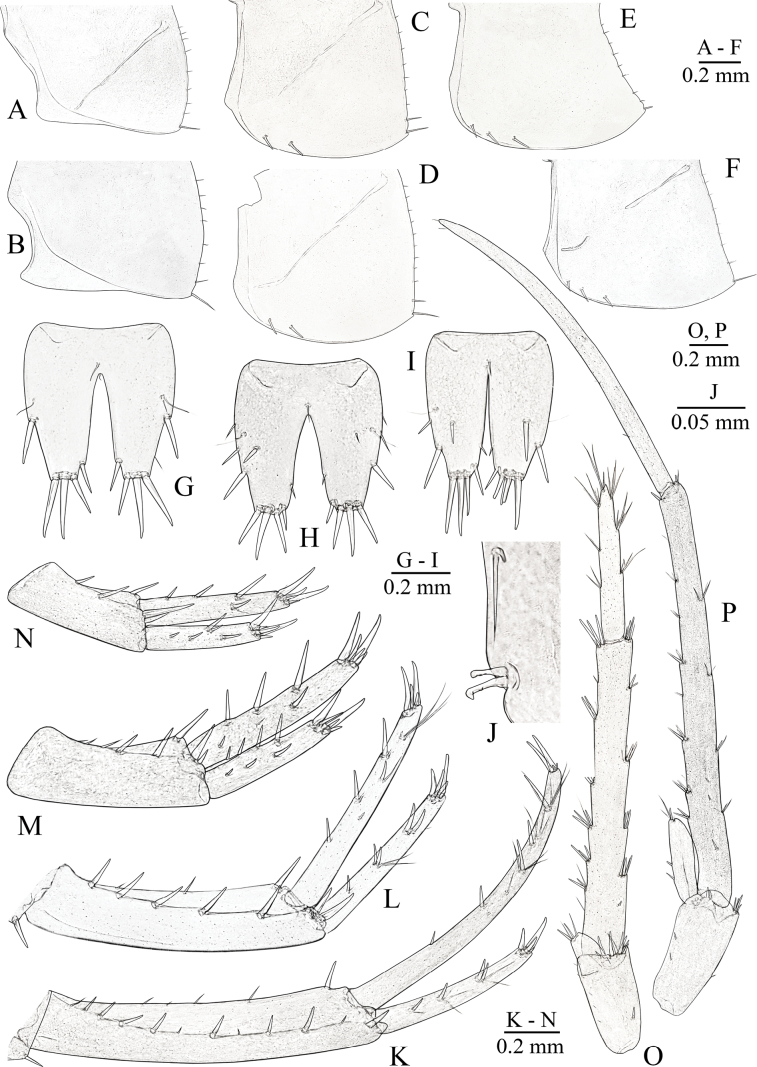
*Niphargus primoricus* sp. nov., holotype ♂ (ZMMU Mb-1299) (A, C, E, I, J, K, M, P), paratype ♀ (ZMMU Mb-1300) (B, D, F, G, L, N, O), ♀ (LEMMI) (H). A–F. Epimeral plates I–III; G–I. Telson; J. Retinacula of pleopod III; K, L. Uropod I; M, N. Uropod II; O, P. Uropod III.

**Figure 8.**
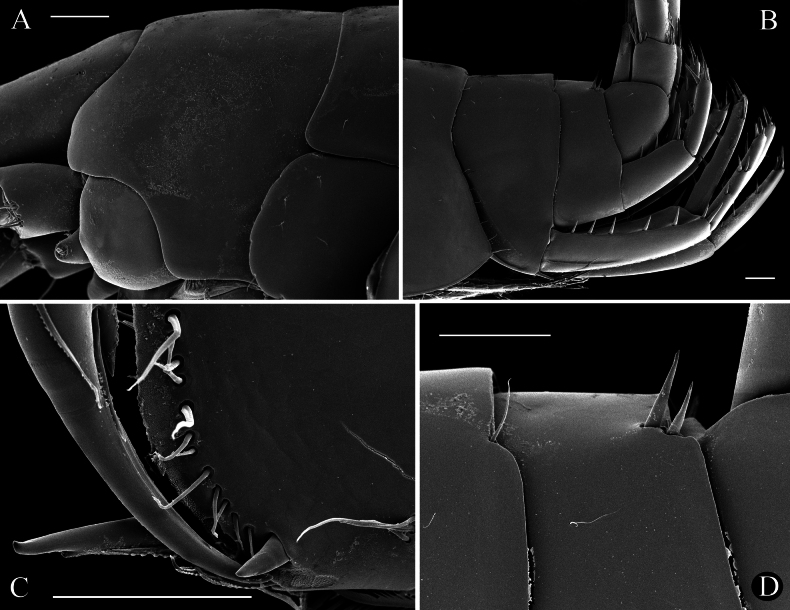
*Niphargus primoricus* sp. nov., ♂ (LEMMI). A. Head; B. Urosomal segments and uropods; C. Distoventral palmar corner of propodus of GnI; D. Dorsal surface of urosomal segments. Scale bars: 100 µm (A–D).

Figs 2, 3, 4, 5, 6, 7, 8

##### Material examined.

• ***Holotype***, ♂ (bl. 8.0 mm) (ZMMU Mb-1299) – Russian Federation, Krasnodar Kray, Sochi urban district, Khostinskiy district, hyporhean zone of the Khosta River within the boundaries of the Khosta village, 43°30'55.3"N, 39°52'10.1"E, coll. I. Marin & D. Palatov, 23.10.2024. • ***Paratypes***, ♂ (bl. 8.0 mm), ♀ (bl. 7.0 mm) (ZMMU Mb–1300) – Russian Federation, Krasnodar Kray, Sochi urban district, Khostinskiy district, hyporhean zone of the mouth of the Khosta River within the boundaries of the Khosta village, 43°30'33.9"N, 39°52'07.0"E, coll. I. Marin & D. Palatov, 23.10.2024.

##### Additional material.

• 3♂♂, 7♀♀ (LEMMI) – same data and locality as holotype; • 3♂♂, 2♀♀ – Russian Federation, Krasnodar Kray, Gelendzhik urban district, Golubaya Bukhta (Blue Bay), 44°34'35.9"N, 37°58'48.3"E, pebble beach near the mouth of Ashamba River, in coastal pebble, coll. I. Marin, 12.07.2023; • 2♂♂, 3♀♀ (bl. 7.0 mm) (LEMMI) – Russian Federation, Krasnodar Kray, Gelendzhik urban okrug, hyporhean zone of the mouth of the Betta River within the boundaries of the Betta village, 44°22'16.67"N, 38°23'48.84"E, coll. I. Marin & D. Palatov, 12.06.2024; • 3♀♀ – Russian Federation, Krasnodar Kray, Tuapse district, hyporheic zone of the mouth of the Agoy River within the boundaries of the Agoy village, 44°08'06.75"N, 39°01'33.5"E, coll. I. Marin & D. Palatov, 07.06.2024; • 1♂, 6♀♀ – Russian Federation, Krasnodar Kray, Tuapse district, hyporhean zone of the mouth of the Nebug River within the boundaries of the Nebug settlement, 44°09'41.6"N, 38°59'52.4"E, coll. I. Marin & D. Palatov, 07.06.2024; • 4♀♀ – Russian Federation, Krasnodar Kray, Tuapse district, Nechepsukho River in the Novomikhaylovsky village, under the pedestrian bridge, 44°14'52.24"N, 38°50'35.37"E, coll. I. Marin & D. Palatov, 08.06.2024; • 2♂♂, 3♀♀ – Russian Federation, Krasnodar Kray, Tuapse district, hyporhean zone of the Agoy River within the boundaries of the village of Agoy, 43°57'45.8"N, 39°16'04.3"E, coll. D. Palatov, 14.07.2020; • 1♂, 6♀♀ – Russian Federation, Krasnodar Kray, Tuapse district, pebble beaches in the valley of the Tu River near its mouth in the Olginka village, 44°11'28.12"N, 38°53'05.52"E, coll. I. Marin & D. Palatov, 08.06.2024; • 2♀♀ (LEMMI) – Russian Federation, Krasnodar Kray, Tuapse district, hyporhean zone of the mouth of the Dederkoy River upstream from the Dederkoy village, 44°04'00.4"N, 39°08'22.8"E, coll. I. Marin & D. Palatov, 13.05.2019; • 4♀♀ – Russian Federation, Krasnodar Kray, Sochi urban district, Lazarevsky district, Makopse River under the railway bridge, near the river mouth, 43°59'34.11"N, 39°12'37.98"E, coll. I. Marin & D. Palatov, 06.06.2024; • 1♀ – Russian Federation, Krasnodar Kray, Sochi urban district, Lazarevsky district, Kuapse River near the Nizhnyaya Mamedka village, 43°57'10.72"N, 39°18'43.1"E, coll. I. Marin & D. Palatov, 05.06.2024; • 2♀♀ – Russian Federation, Krasnodar Kray, Sochi urban district, Lazarevsky district, Ashe River mouth, 43°57'28.8"N, 39°15'41.1"E, coll. I. Marin & D. Palatov, 05.06.2024; • 3♂♂, 12♀♀ – Russian Federation, Krasnodar Kray, Sochi urban district, Khostinskiy district, hyporhean zone of the mouth of the Khosta River within the boundaries of the Khosta village, 43°30'33.9"N, 39°52'07.0"E, coll. I. Marin & D. Palatov, 15.05.2019; • 3♀♀ – Russian Federation, Krasnodar Kray, Sochi urban district, Lazarevsky district, Psezuapse River near the Lazarevskoye village, 200 m upstream from the mouth, 43°53'57.35"N, 39°20'03.97"E, coll. I. Marin & D. Palatov, 03.06.2024.

##### Diagnosis.

Head with small yellow pigmented spots on anterior lobe. Posteroventral corners of epimeral plates I–III bluntly rounded. Urosomite I unarmed; urosomite II with one simple seta on each side dorsolaterally; urosomite II with two strong spines on each side dorsolaterally. Propodus of gnathopods I and II subtrapezoidal, with its width shorter than depth; dactylus with numerous simple setae along outer margin. Dactyli of pereopods III–VII with one small additional median spine and one median short plumose seta at outer margin. Rami of uropod I unequal in size: endopodite ~ 1.8× longer than exopodite in mature males, and 1.2–1.25× longer than exopodite in small males and females, with tufts of well-seen long curved setae in both sexes; exopodite of uropod III differ in males and females, significantly longer in males. Pleopods with two hooks in retinacles. Telson with two, three, or four medium-sized distal spines on each lobe and one or two lateral spines, accompanied by two small plumose setae, and its dorsal surface with none or one small or medium submarginal spine on each side and none or one small mesial seta.

##### Description

**(based on holotype male, ZMMU Mb-1299).** Body depigmented, stygomorphic, moderately slender (see Fig. 2).

***Head*** (Fig. 2B): length is ~ 7.5% of body length; rostrum and pigmented spots on anterior lobe absent, with bluntly produced anteroventral lobes and excavated anteroventral sinus. Eyes cornea absent, but small pigmented yellow spots well seen on head (Fig. 2B).

***Pereon***: pereonites I–VII without setae, smooth.

***Pleosoma***: pleonites I–III with several short marginal setae on each posterodorsal margin.

***Epimeral plates*** (Fig. 7A, C, E). Posteroventral corners of epimeral plates I and II rounded (Fig. 7A, C), epimeral plate III with posteroventral corner posteriorly produced, nearly right-angled (Fig. 7E). Epimeral plate I (Fig. 7A): posterior margin convex, ventral margin slightly convex; without spines along ventral margin; with eight setae along posterior margin; posteroventral angle with one strong seta. Epimeral plate II (Fig. 7C): posterior and ventral margin convex; with two spiniform setae along ventral margin; eight setae along posterior margin, one of which strong and close to posteroventral angle; posteroventral angle with one strong seta. Epimeral plate III (Fig. 7E): posterior margin almost straight slightly convex, ventral margin slightly convex; with three spiniform setae along ventral margin; with eight setae along posterior margin; posteroventral angle with one strong seta.

***Urosomites*** (Fig. 8B, D): urosomite I with one long simple seta on each side dorsolaterally, with one posteroventral long spine-like near basis of uropod I; urosomite II with two simple strong spines each side dorsolaterally; urosomite III unarmed.

***Coxae***: coxal plate I (Fig. 5D) oval in shape, with rounded anteroventral margin, armed with 11 setae, width/depth ratio 1/1.3; coxal plate II (Fig. 5G) close to quadrate, with rounded anteroventral margin, armed with 8 setae, width/depth ratio ~ 1/1.1; width/depth ratio of coxal plates III and IV (Fig. 5A, C) 1/0.9 and 1/1, respectively; anterior and ventral margins of coxal plates III–IV with ten and seven setae each, respectively; with rounded anteroventral corners; coxal plates V and VI (Fig. 5E, G) with large lobes anteriorly, provided with six and four setae, respectively; posterior margins with three and one setae each, respectively; coxal plate VII (Fig. 5I) semicircular and with one strong and one simple setae on posterior lobe; coxal gills II–VI ovoid of which II and IV more elongated, length ratio of gills/bases of pereopods ~ 0.72/1, 0.84/1, 0.97/1, 0.83/1 and 0.73/1, respectively.

***Antenna I*** (Fig. 3A): slender, ~ 48–50% of body length; peduncular articles moderately slender, ratio 1/0.8/0.31; flagellum with 28 articles, most of them with two short aesthetascs each; accessory flagellum short, bi-articulated (Fig. 3B), with several long apical setae; length ratio of antennas I/II in range of 1/0.60–0.75.

***Antenna II*** (Fig. 3C): peduncular articles moderately stout, with several long setae along ventral margin, dorsal setae shorter than inner ones; flagellum relatively short with relatively short setae, consisting in males with 11 articles; peduncular articles II and III short, approx. as long as wide, peduncular articles IV and V slender, ~ 5× and 6× longer than wide, respectively, length ratio of 0.98–1/0.90–0.95; flagellum ~ 0.45–0.48 of length of peduncular articles IV+V.

***Labrum*** (upper lip) (Fig. 4A) typical.

***Labium*** (lower lip) (Fig. 4B): with entire, oblong oval outer lobes and well-developed smaller inner lobes.

***Mandible*** (Fig. 4C, E): Left mandible (Fig. 4C): incisor process with five teeth, lacinia mobilis with four teeth; with row of nine serrated setae between lacinia and molar process (Fig. 4D); mandibular palp proximal article without setae, ~ 2× longer than wide; mandibular article II/III (distal) ratio 1/0.3; article II with 12–13 simple setae; distal article with group of five A-setae; four groups of B-setae; 22 D-setae and six E-setae. Right mandible (Fig. 4E): incisor process with four blunt teeth, lacinia mobilis bifurcate distally, with row of nine serrated setae between lacinia and molar process (Fig. 4F).

***Maxilla I*** (Fig. 3G): inner lobe with three simple distal setae, outer lobe with seven robust spines (2 spines with 2 strong lateral teeth and 3 spines with 1 strong lateral tooth (1–0–1–0–1–2–2) (Fig. 3H)); palp bi-articulated, distal article with four simple setae distally.

***Maxilla II*** (Fig. 4I): both plates with numerous long distal simple setae, outer lobe with row of fine setae along outer margin.

***Maxilliped*** (Fig. 4J): inner plate short, with four distal robust setae intermixed with six distal simple setae; outer plate reaching half of palpal article II and bearing row of 20 or 21 distolateral spines and distal setae; palpal segment III with single median bundle of setae, along with single bundles of setae located at distal edge on both its internal and external surfaces; furthermore, large simple seta is situated at center of external margin of article; palpal article IV with one median seta at outer margin; nail shorter than pedestal, with small seta near basis.

***Gnathopod I*** (Fig. 3D): basis elongated, width/length ratio ~ 0.37/1, with distal part greatly expanded, with long simple setae along anterior and posterodistal margins; ischium approx. as long as wide, with group of posterodistal setae; merus subquadrate, equal in size to ischium; carpus approx. as long as wide, ~ 0.40× of length of basis and 0.46× of length of propodus, with single distal group of setae anteriorly, with transverse rows of setae along posterior margin and row of setae posterolaterally; propodus subtrapezoidal, ~ 1.25× longer than wide, setose, with six rows of setae at posterior margin, anterior surface with three groups of total from four to six setae each in addition to anterodistal group of five or six setae; several groups of short setae on inner surface; palmar corner armed with long spiniform palmar seta, two serrated spiniform setae, single supporting spiniform seta on inner surface (Fig. 3E); dactylus with six setae along anterior margin, some of them are grouped in pairs, and with row of short setae along inner surface; length of nail 0.35× of total length of dactylus.

***Gnathopod II*** (Fig. 3G): basis width/length ratio ~ 0.31/1, with distal part greatly expanded, with long simple setae along anteriorly on posterior and posterodistal margins; ischium approx. as long as wide, with three posterodistal setae; merus subquadrate, slightly longer than ischium, with eight posterodistal setae; carpus 0.57× of length of basis and 0.82× of length of propodus, with distal group of setae anteriorly, few transverse rows of setae along posterior margin and row of setae posterolaterally; propodus subtrapezoidal, ~ 1.14× longer than wide, setose, larger than propodus (palm) of GnI (GnI/II as 0.88/1), posterior margin with six rows of setae, anterior surface with two groups of setae in addition to six or seven anterodistal setae, with several groups of setae on inner surface, palmar corner with one strong palmar spiniform seta, one supporting spiniform seta on inner surface and two denticulated thick spiniform setae on outer side (Fig. 3H); dactylus with five setae along anterior surface some of them are grouped in pairs and few short setae along inner surface; length of nail ~ 0.34× of total length of dactylus.

***Pereopods III and IV*** (Fig. 5A, C) almost similar in size and shape; basis ~ 4.1–4.3× as long as wide, with posterior margin bearing long marginal setae, with distoventral group of setae; ischium short, subquadrate, approx. as long as wide, with distoventral group of setae; merus ~ 4.3–4.4× longer than wide, with slender simple setae along anterior and posterior surfaces; carpus/propodus ratio ~ 0.95–0.96/1; propodus with four groups of spines along ventral margin; dactylus (Fig. 5B, D) relatively stout, curved, sharp distally, with one small additional posterior median spine and one median short plumose seta at outer margin; length of nail ~ 0.4× of total length of dactylus.

***Pereopods V–VII*** (Fig. 5E, G, I): length ratio of PpV/VI/VII close to 1/1.42/1.48.

***Pereopod V*** (Fig. 5E): basis almost rectangular, with length/width ratio ~ 1/0.61, with explicit posteroventral lobe; with facial setae; posterior margin almost straight with row of 15 slender marginal setae; anterior margin convex, with row of six slender marginal setae, which distinctly longer than posterior ones, and group of setae in distal part; ischium subquadrate, approx. as long as wide, with long posterodistal setae; merus ~ 3.0× longer than wide, with three slender spines along anterior surface and with two spine on posterior surface; carpus ~ 4.6× as long as wide, approx. as long as merus, with several bunches of slender spines; propodus slender, ~ 6.0–6.3× as long as wide, with several bunches of slender spines; dactylus (Fig. 5F) with one small additional posterior median spine and one median short plumose seta at outer margin; length of nail close to 0.27–0.3× of total length of dactylus.

***Pereopod VI*** (Fig. 5G): basis almost rectangular, with length/width ratio ~ 1/0.57, with distinct posteroventral lobe and straight posterior margin bearing row of 16 short marginal setae, anterior margin convex, with row of five longer marginal setae, which distinctly longer than posterior ones, and group of setae in anterodistal part; ischium approx. as long as wide, subquadrate, with long posterodistal setae; merus ~ 3.4× longer than wide, with several bunches of short spines along anterior and posterior margins; carpus ~ 5.9× as long as wide, with group of spines intermixed with single short setae; propodus slender, ~ 8.5× as long as wide, with several group of short spines; dactylus (Fig. 5H) slender, with single small additional posterior median spine and single short median plumose seta at outer margin; length of nail close to 0.3× of total length of dactylus.

***Pereopod VII*** (Fig. 5I) similar to PVI: basis almost rectangular, with length/width ratio ~ 1/0.65, with distinct posteroventral lobe and straight posterior margin bearing row of 13 short marginal setae, anterior margin convex, with row of six longer marginal setae, which distinctly longer than posterior ones, and group of setae in anterodistal part; ischium slightly wider than long, subquadrate, with long posterodistal setae; merus ~ 2.7× longer than wide, with several bunches of short spines along anterior and posterior margins; carpus ~ 5.1× as long as wide, with group of spines intermixed with single short setae; propodus slender, ~ 10.0× as long as wide, with several group of short spines; dactylus (Fig. 5J) slender, with one small additional posterior median spine and one short median plumose seta at outer margin; length of nail close to 0.29× of total length of dactylus.

***Pleopods***: pleopod I with basal segments without setae, with two coupling hooks in retinacula and long simple setae above hooks (Fig. 7J); outer and inner rami with 14 and 17 segments, respectively; pleopod II with basal segments without setae, with two coupling hooks in retinacula; outer and inner rami with 13 and 14 segments, respectively; pleopod III with basal segment with two or three long simple setae, with two coupling hooks, with long simple setae above hooks in retinacula; outer and inner rami with 11 and 12 segments, respectively.

***Uropod I*** (Fig. 7K): protopodite ~ 5.5× longer than wide, with dorso-external row of 10–11 spines or spiniform setae, and dorsointernal row of with four–six thin spines; length ratio of protopodite/endopodite/exopodite ~ 1/0.93/0.49; endopodite elongated, not paddle-like, with four or five dorsolateral spines accompanied by groups of long bristles and with two long and two short apical spines; exopodite with four dorsolateral spines, accompanied by groups of long bristles and four apical spines.

***Uropod II*** (Fig. 7M): protopodite ~ 3.2× as long as wide, with dorso-external row of four or five spines or spiniform setae, and dorso-internal row of one or two thin spines; length ratio of protopodite/endopodite/exopodite ~ 1/0.99/0.75; rami with dorsal, lateral and apical slender spines: endopodite with three dorsal, one dorsolateral and five apical spines; exopodite with four dorsal, three dorsolateral and five apical spines.

***Uropod III*** (Fig. 7P): ~ 0.45× of body length; protopodite ~ 2.5× as long as wide, with one small external seta, two small internal setae, and four or five apical spiniform setae; rami unequal, endopodite short, ~ 10–11× shorter than exopodite, with two small setae laterally and two or three apical setae; proximal article ~ 12× longer than wide, with seven or eight groups of thin-flexible, plumose and spiniform setae along inner and outer margins; distal article ~ 19× longer than wide, ~ 0.86× of length of proximal article, with two simple setae apically.

***Telson*** (Fig. 7I): slightly longer then wide, ~ 1.1× as long as wide; cleft ~ 0.72–0.8× of length of telson; margins weakly rounded, narrowing apically; with four or five medium-sized distal spines on each lobe and from one to three lateral spines, accompanied by one or two plumose setae on each side; dorsal surface with one small or medium submarginal spine on each side and with none or one small mesial seta.

**Figure 9.**
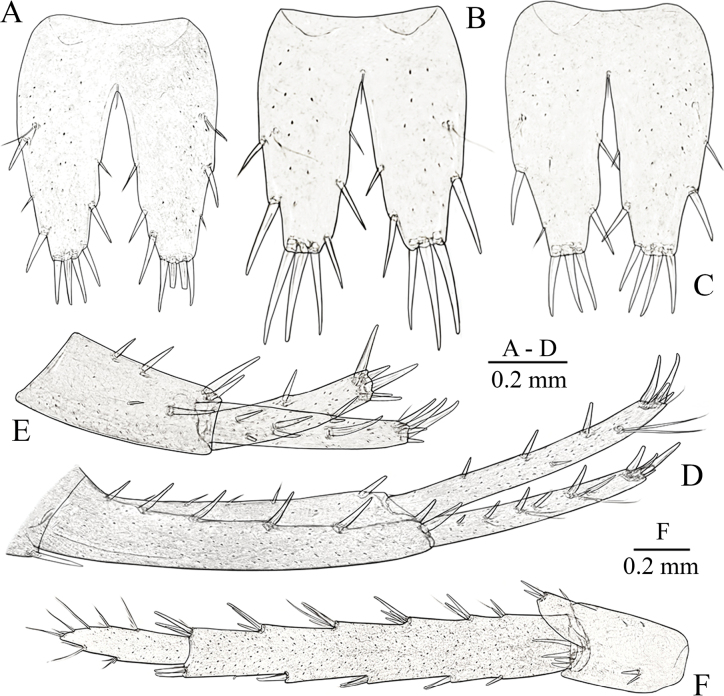
*Niphargus primoricus* sp. nov., ♂♂ (LEMMI). A–C. Telson; D. Uropod I; E. Uropod II; F. Uropod III.

**Females** (Figs 3F, I, 6, 7B, D, F–H, L, N, O) and small-sized males (Fig. 9). Females are very similar to males in size. Morphologically small-sized males close to females, especially in shape of uropods I–III (see Fig. 9). Gnathopods I and II (Fig. 3F, I) and ambulatory pereiopods (Fig. 6) are almost similar to males. Epimeral plate I (Fig. 7B): posterior margin convex, ventral margin slightly convex; without spines along ventral margin; with seven setae along posterior margin; posteroventral angle with one strong seta; epimeral plate II (Fig. 7D): posterior and ventral margin convex; with two spiniform setae along ventral margin; ten setae along posterior margin, one of which strong and close to the posteroventral angle; posteroventral angle with one strong seta; epimeral plate III (Fig. 7F): posterior margin almost straight slightly convex, ventral margin slightly convex; with three spiniform setae along ventral margin; with eight setae along posterior margin; posteroventral angle with one strong seta. Length of PVII to the total body length ~ 39–40% in males and 53–55% in females, respectively (see Fig. 3). Length of the protopodite uropod I is much greater than the width in males than in females, ~ 5.5× and 5.0×, respectively; length ratio of protopodite/ endopodite/ exopodite ~ 1/0.93/0.49 in large males (Fig. 7K) and 1/0.78/0.60 in small males (Fig. 9D) and females (Fig. 7L), respectively. Protopodite of uropod II is shorter in females and small-sized males vs large-sized males, ~ 2.4× as long as wide in large males (Fig. 7M) and ~ 3.0× in females (Fig. 7N) and small-sized males (Fig. 9E), respectively; length ratio of protopodite/endopodite/exopodite ~ 1/1.06/0.84. Uropod III different in large-sized males vs females and small-sized males, ~ 0.43× of body length in large-sized males (Fig. 7P) and 0.30× in females (Fig. 7O) and small-sized males (Fig. 9F); distal article ~ 19× longer than wide in large-sized males (Fig. 7P) and 6.8× in females (Fig. 7O) and small-sized males (Fig. 9F). Uropod III (Fig. 7O): protopodite ~ 1.9× as long as wide, with from two to five thin setae laterally and from five to seven spiniform setae apically; rami unequal, endopodite short, ~ 10× shorter than exopodite, without seta laterally and two or three spiniform setae apically; distal article 0.5× of length of proximal article, with three or four groups of thin-flexible setae along each margin and group of simple setae apically; proximal article ~ 8.0–9.0× longer than wide, with four groups of spiniform setae along outer margin and five groups of spiniform setae along inner margin. Telson (Fig. 7G–I) similar both in males and females; cleft ~ 0.65–0.7× of length of telson.

##### Coloration.

Body, appendages, and internal organs are whitish or yellowish characteristic to other stygobiotic representatives of the genus *Niphargus* (see Fig. 2).

##### Body size.

The largest collected male has bl. 8.0 mm; the largest collected female has bl. 15.5 mm.

##### GenBank

**(NCBI) accession numbers.**PX233131 (holotype ♂, ZMMU Mb-1299), PX233133 (paratype ♀, ZMMU Mb-1300), PX233131–PX233153.

##### Habitat.

All specimens of *Niphargus primoricus* sp. nov. were collected from the deep layers of pebble beach habitats along the northern Black Sea coastline (Fig. 1A). This species inhabits freshwater environments, such as small rivers, streams, and springs, which ooze in the lower layers of coastal pebbles near the edge of the sea (Fig. 1C–F). These biotopes are common along the northern coast of the Black Sea, alternating with rocky outcrops and small sandy beaches. Also, several individuals of this species have been found in hyporhean habitats under thick layers of pebbles in the small mountain river Kuapse (near the village of Nizhnyaya Mamedka), which flows into the Black Sea.

##### Distribution.

The species is currently found along the northern coastline of the Black Sea, from the mouth of the Ashamba River (44°34'35.9"N, 37°58'48.3"E) to the Khosta River (43°30'33.9"N, 39°52'07.0"E) (the type locality) (see Fig. 1), with the most distant localities separated for 190 km.

##### Etymology.

The new species is named after its unique habitat and distribution along the northern Black Sea coastline; *primoricus* (Russian) meaning “living close to the sea shore”.

##### Taxonomic remarks.

*Niphargus primoricus* sp. nov. clearly belongs to the European “*stygius*–*longicaudatus*” species group (Fig. 1) according to the molecular genetic analysis, closely related to *Niphargus tarkhankuticus* Marin, Turbanov, Prokopov & Palatov, 2022 from Tarkhankut Peninsula (Crimea), as well as *N. longicaudatus* (A. Costa, 1851), *N. aitolosi* Ntakis, Anastasiadou, Zakšek & Fišer, 2015, *N. cvijici* S. Karaman, 1950, *N. frasassianus* G. Karaman, Borowsky & Dattagupta, 2010, *N. pasquinii* Vigna-Taglianti, 1966, *N. sibillianus* G. Karaman, 1984, *N. sodalis* G. Karaman, 1984, *N. timavi* S. Karaman, 1954, and *N. versluysi* S. Karaman, 1950, known in the Apennine and Balkan Peninsulas (see Karaman 1986, 1989; Marin et al. 2022). These species have distally bluntly produced anterodistal lobe of the head; the presence of two dorsolateral spines on urosomite III; the presence of two hooks in retinacles; uropod I with different sized rami, with endopodite ramus usually larger than exopodite; rami of uropod I with characteristic thin-flexible setae; and bluntly rounded posteroventral corners of epimeral plates.

Together with Crimean *N. tarkhankuticus* and two undescribed species from Crimea (Staryi Krym) and northern Türkiye (Akgüney, Sinop) (IM, unpublished data) (see Fig. 1), the new species forms a geographically local Pontic (of the Black Sea area) group of species, which has been named as the “*tarkhankuticus*” ingroup (see Marin et al. 2022). However, the new species can be separated from *N. tarkhankuticus* by several minute morphological features, such as 1) almost straight posterior margin of epimeral plate II and triangularly produced posterolateral angle of epimeral plate III; 2) significantly longer distal spines on lobes of telson; and 3) strongly spinulated protopodites of uropods I and II. At the same time, morphological similarity in most other morphological features clearly indicates a very close phylogenetic relationship between these species.

To distinguish from the related European species, namely *N. longicaudatus*, *N. aitolosi*, *N. cvijici*, *N. frasassianus*, *N. pasquinii*, *N. sibillianus*, *N. sodalis*, *N. timavi* and *N. versluysi*, see Marin et al. (2022), where a detailed morphological comparison is given with *N. tarkhankuticus*, which is phylogenetically and morphologically very close to the new species (see above)..

##### Molecular phylogenetic approach.

The molecular genetic analysis (Fig. 1B) clearly confirmed the monophyly (Bayesian–PP = 1.00; ML–BS = 95%) of the studied lineage, including *Niphargus primoricus* sp. nov., *N. tarkhankuticus*, and two undescribed species from the coastal habitats of the Black Sea (see Fig. 1B; Table 2).

**Table 2.** Uncorrected pairwise genetic (COI mtDNA) distances (*p*-distances) (substitutions per 100 nucleotides) (± S.E.) between *Niphargus primoricus* sp. nov. (*n* = 17) and other relative congeners.

Species (or locality for undescribed species)	*p*-distances ± S.E.
*Niphargus* sp. – Türkiye: Akgüney, Sinop (*n* = 2)	0.121±0.026
*Niphargus tarkhankuticus* (*n* = 6)	0.128±0.027
*Niphargus* sp. – Crimean Peninsula: Staryi Krym (*n* =2)	0.135±0.027
*N. pasquinii* (*n* = 4)	0.181±0.038
*N. cvijici* (*n* = 1)	0.184±0.039
*N. frasassianus* (*n* = 3)	0.185±0.039
*N. timavi* (*n* = 3)	0.188±0.039
*N. sodalis* (*n* = 1)	0.191±0.040
*N. longicaudatus* (*n* = 6)	0.193±0.040
*N. aitolosi* (*n* = 3)	0.194±0.041
*N. sibillianus* (*n* = 3)	0.206±0.044
*N. versluysi* (*n* = 3)	0.209±0.044

The interspecific genetic differences between the studied related *Niphargus* species mostly vary from 12% to 13% (see Table 1).

The intraspecific genetic divergence between the individuals from recently discovered locations (the most remote of them are separated by a distance of 190 km) (see Table 1) of *Niphargus primoricus* sp. nov. (*n* = 17) is very low, with the interspecific uncorrected pairwise distances (*p*-distances) estimated as ~ 0.8% (0.008±0.001 substitutions per 100 nucleotide positions).

##### ZooBank taxon ID.

The electronic version of this article in Portable Document Format will represent a published work according to the International Commission on Zoological Nomenclature (ICZN), and hence the new names contained in the electronic version are effectively published under that Code from the electronic edition alone. This published work and the nomenclatural acts it contains have been registered in ZooBank, the online registration system for the ICZN. The ZooBank Life Science Identifiers (LSID) for this publication is: https://zoobank.org/D09561CD-8A55-4127-999B-FEC5C07237D0. The online version of this work is archived and available from the following digital repositories: ZooKeys, PubMed Central and CLOCKSS. The LSID for the new species, *Niphargus primoricus* sp. nov., is https://zoobank.org/3ED10492-D4D5-420A-A2EF-9511C8E17C2C.

## ﻿Discussion

The newly discovered species belong to the separate lineage, which we suggest calling “*tarkhankuticus*” ingroup. The ingroup currently includes *N. tarkhankuticus*, *Niphargus primoricus* sp. nov., and two undescribed species from the coastal habitats of the Black Sea (see Fig. 1B). The phylogenetic analysis also supports the relationship of this ingroup with some representatives of the European “*longicaudatus*” species group, such as *N. frasassianus*, *N. cvijici*, *N. sodalis*, *N. pasquinii*, *N. sibillianus*, *N. longicaudatus*, and *N. versluysi* (see Fig. 1B). The genetic separation of the “*tarkhankuticus*” ingroup from its European counterpart was previously calculated (see Marin et al. 2022) and was estimated to have occurred during the Late Miocene, approximately 11–10 Mya, and was probably influenced by the fragmentation of the Eastern Paratethys into distinct basins (e.g., Popov et al. 2004, 2006). The interspecific genetic divergence (see Table 2) justifies the long isolation of these species during a period of ca 5.0–3.6 Mya (sensu Lefébure et al. 2006, 2007; Copilaş-Ciocianu and Petrusek 2018; Guy-Haim et al. 2018; Copilaş-Ciocianu et al. 2019). In the historical record of the Ponto-Caspian region, this period is likely associated with a change in the salt content of the Black Sea, as well as the Messinian Salinity Crisis (5.96–5.33 Mya) and a significant decrease in the level of the Black Sea (e.g., Müller et al. 1999; Popov et al. 2004, 2006; Neubauer et al. 2015), that may have led to the separation of a single species into separate, isolated populations.

The discovery of *Niphargus primoricus* sp. nov. reveals the new type of habitats in a rather unusual biotope – coastal pebble beaches – from where species of *Niphargus* had never been previously reported. Careful studies of the upstream hyporhean and adjacent marine biotopes have not detected this species. We can therefore conclude with confidence that it lives exclusively in freshwater resources such as springs, streams, and small coastal rivers that flow in deep layers of pebble beaches at the seashore. Due to the fact that this species lives in waters that are in between the sea and fresh water, it is likely that the salinity in these waters can vary greatly. We did not measure salinity at this time, but we will probably do so in a more detailed study of this species’ ecology and other amphipod species that live in coastal areas. At the same time, there have been reports of other *Niphargus* species found in brackish water (e.g., Sars 1894; Sket 1977; Kokalj et al. 2022), which may have arrived there through leaching from underground freshwater sources.

This species also has a significant distribution (currently known to be almost 190 kilometers) along the Black Sea coastline and a low genetic divergence between the individuals from different locations (see Table 1), much lower than in other Ponto-Caspian and Caucasian species studied (e.g., Copilaş-Ciocianu et al. 2014, 2017, 2018; Marin 2019; Palatov and Marin 2021; Marin et al. 2021b, 2022, 2023b). As we noted above, this biotope also houses specific species of gammarid amphipods, *Dursogammarus dromaderus* and *Litorogammarus dursi* (Amphipoda: Gammaridae) (see Marin and Palatov 2022; Marin et al. 2023a), whose biology has not been studied, and it is unknown how they spread along the coast. Interestingly, to date, none of the species found in these pebble beach habitats have been found in neighboring marine biotopes, possibly indicating their association with freshwaters river flowing through the lower layers of pebbles on the beach. Due to the lack of common river basins along the northern coast of the Black Sea and even large rivers, whose flow during the maximum flood could theoretically extend over a significant distance, the main question arises: how this species spreads and how connection (gene drift) is maintained between individuals from different locations?

Similar distribution, along large rivers or around the coast of large water basins, is known for a number of epigean species, for example *N. hrabei* S. Karaman, 1932, *N. valachicus* Dobreanu & Manolache, 1933, and *N. potamophilus* Birštein, 1954 (Copilaş-Ciocianu et al. 2014, 2017, 2018; Palatov and Marin 2021; Marin and Palatov 2023). Caucasian *Niphargus bzhidik* Marin, Krylenko & Palatov, 2021 shows a distribution in the drainage valleys (basins) of several neighboring small mountain rivers, namely Pshada (Skupkova Schel only), Vulan (with a tributary of Tekos River), Teshebs and Bzhid, flowing into the Black Sea in the Tuapse area of the Krasnodar region, Russia (Marin et al. 2021b). These species also show a low level of genetic divergence between highly geographically isolated populations (Copilaş-Ciocianu et al. 2014, 2017, 2018; Palatov and Marin 2021; Marin and Palatov 2023). For example, the spread of epigean species from the Danube basin has been probably linked to seasonal river flooding, which represent the passive long-range dispersal events (van Leeuwen et al. 2013; Copilaş-Ciocianu et al. 2018). However, this hypothesis cannot explain the presence of these species on the eastern coast of the Black Sea and Caspian Basin. These species probably settled in the Black Sea basin shortly after the Last Glacial Maximum (30–25 Kya). At that time, the area of the Black and Azov seas, including the mouths of the Dnieper, Dniester, Danube, and Don rivers, was a freshwater lake separated from the World Ocean (Ryan et al. 1997; Copilaș-Ciocianu et al. 2017, 2018; Parvizi et al. 2019; Jablonski et al. 2019). Recently (ca 9–7 Kya), it was separated by a flood of salty water from the Mediterranean Sea and became a sea (Federov 1971; Bahr et al. 2006; Georgievski and Stanev 2006). It is also possible that the observed pattern of the settlement of several species in the Caspian Sea basin formed quite recently, perhaps during the Late Pleistocene period (129–11.7 Kya). During this time, water levels and salinity in the Black Sea and Caspian Sea differed significantly, and there were several instances of direct communication between the two bodies of water (Badertscher et al. 2011). Molecular genetic data from various groups of aquatic invertebrates suggest the existence of an ancient refugium in the deltas of the Sea of Azov during the Plio–Pleistocene period, where genetically stable populations or species have been preserved for a long period of time (Tomilova et al. 2020). The separation of various phylogenetic lineages in separate basins of the rivers of the Azov Sea probably occurred in the late Pliocene (ca 3.6–2.6 mya), which probably contributed to the change in the boundaries of freshwater basins in the Ponto-Caspian region (Tomilova et al. 2020).

At the same time, it is worth noting that *Niphargus primoricus* sp. nov. differs from the previous examples of epigean *Niphargus* species in its lifestyle and stygomorphic morphology. Specifically, it lacks visible eyes and but have small pigmented yellow spots (probably photosensitive) on its head and lives in deep layers of pebbles, obviously never appearing on the surface. In contrast, epigean species usually have eyes and/or pigmented spots and inhabit surface water reservoirs, being probably not adapted to survive in the stygobiotic environments. Thus, unlike the known underground stygobiotic species, *Niphargus primoricus* sp. nov. has a unique biotope that is not represented by a network of connected underground reservoirs where it can move and spread. Instead, it lives in isolated freshwater streams along the sea coastline, probably unable to survive and thrive in salty marine waters.

We believe that the unusual distribution pattern of stygobiotic *Niphargus* species in the Ponto-Caspian region, as presented in this study, will help to uncover and understand historical phylogeographic processes in this area. We also believe that the study of the biology of this species will shed light on the biology and patterns of distribution of other species living in similar coastal biotopes, the further study of its biology and probable ways of distribution will also help to understand the biology of other amphipod species living in this biotope. The conducted research clearly showed that the coastal part of the Black Sea and the adjacent pre-Caucasian river/land areas harbors a significant undescribed diversity, and that the transitional sea/river brackish biotopes are important reservoirs of the endemicity.

## Supplementary Material

XML Treatment for Niphargus primoricus

## ﻿Additional information

### Use of AI

No use of AI was reported.

### Author ORCIDs

Ivan N. Marin https://orcid.org/0000-0003-0552-8456

Dmitry M. Palatov https://orcid.org/0000-0002-8826-9316

### Data availability

All of the data that support the findings of this study are available in the main text.

## References

[B1] Badertscher S Fleitmann D Cheng H Edwards RL Göktürk OM Zumbühl A Leuenberger M Tüysüz O (2011) Pleistocene water intrusions from the Mediterranean and Caspian seas into the Black Sea. Nature Geoscience 4(4): 236–239. 10.1038/ngeo1106

[B2] Bahr A Arz HW Lamy F Wefer G (2006) Late glacial to Holocene paleoenvironmental evolution of the Black Sea, reconstructed with stable oxygen isotope records obtained on ostracod shells. Earth and Planetary Science Letters 241(3-4): 863–875. 10.1016/j.epsl.2005.10.036

[B3] Bandelt HJ Forster P Röhl A (1999) Median-Joining networks for inferring intraspecific phylogenies. Molecular Biology and Evolution 16(1): 37–48. 10.1093/oxfordjournals.molbev.a026036 10331250

[B4] Bardjadze S Asanidze Z Gavashelishvili A Soto-Adames FN (2018) The hypogean invertebrate fauna of Georgia (Caucasus). Zoology in the Middle East 65(237): 1–10. 10.1080/09397140.2018.1549789

[B5] Cane MA Braconnot P Clement A Gildor H Joussaume S Kageyama M Khodri M Paillard D Tett S Zorita E (2006) Progress in paleoclimate modeling. Journal of Climate 19: e5031e5057. 10.1175/JCLI3899.1

[B6] Chertoprud ES Palatov DM Borisov RR Marinskiy VV Bizin MS Dbar RS (2016) Distribution and a comparative analysis of the aquatic invertebrate fauna in caves of the western Caucasus. Subterranean Biology 18: 49–70. 10.3897/subtbiol.18.8648

[B7] Chertoprud EM Palatov DM Vinarski MV (2021) Revealing the stygobiont and crenobiont Mollusca biodiversity hotspot in the Caucasus: Part III. Revision of stygobiont microsnails (Mollusca: Gastropoda: Hydrobiidae) from the Russian part of Western Transcaucasia, with the description of new taxa. Zootaxa 5005(3): 257–275. 10.11646/zootaxa.5005.3.2 34811261

[B8] Copilaş-Ciocianu D Petrusek A (2015) The southwestern Carpathians as an ancient centre of diversity of freshwater gammarid amphipods: Insights from the *Gammarus fossarum* species complex. Molecular Ecology 24(15): 3980–3992. 10.1111/mec.13286 26096651

[B9] Copilaş-Ciocianu D Grabowski M Pârvulescu L Petrusek A (2014) Zoogeography of epigean freshwater Amphipoda (Crustacea) in Romania: Fragmented distributions and wide altitudinal variability. Zootaxa 3893(2): 243–260. 10.11646/zootaxa.3893.2.5 25544521

[B10] Copilaș-Ciocianu D Fišer C Borza P Balázs G Angyal D Petrusek A (2017) Low intraspecific genetic divergence and weak niche differentiation despite wide ranges and extensive sympatry in two epigean *Niphargus* species (Crustacea: Amphipoda). Zoological Journal of the Linnean Society 181(3): 485–499. 10.1093/zoolinnean/zlw031

[B11] Copilaş-Ciocianu D Fišer C Borza P Petrusek A (2018) Is subterranean lifestyle reversible? Independent and recent large-scale dispersal into surface waters by two species of the groundwater amphipod genus *Niphargus*. Molecular Phylogenetics and Evolution 119: 37–49. 10.1016/j.ympev.2017.10.023 29108937

[B12] Copilaş-Ciocianu D Sidorov D Gontcharov A (2019) Adrift across tectonic plates: Molecular phylogenetics supports the ancient Laurasian origin of old limnic crangonyctid amphipods. Organisms, Diversity & Evolution 19(2): 191–207. 10.1007/s13127-019-00401-7

[B13] Culver DC Pipan T (2009) The Biology of Caves and Other Subterranean Habitats. Oxford University Press, Oxford, United Kingdom, 256 pp.

[B14] Delić T Trontelj P Zakšek V Fišer C (2016) Biotic and abiotic determinants of appendage length evolution in a cave amphipod. Journal of Zoology 299: 42–50. 10.1111/jzo.12318

[B15] Delić T Trontelji P Rendŏs M Fišer C (2017) The importance of naming cryptic species and the conservation of endemic subterranean amphipods. Scientific Reports 7(1): e3391. 10.1038/s41598-017-02938-z PMC5469755 28611400

[B16] Eme D Zagmajster M Delić T Fišer C Flot J-F Konecny-Dupré L Pálsson S Stoch F Zakšek V Douady CJ Malard F (2017) Do cryptic species matter in macroecology? Sequencing European groundwater crustaceans yields smaller ranges but does not challenge biodiversity determinants. Ecography 41(2): 424–436. 10.1111/ecog.02683

[B17] Esmaeili-Rineh S Sari A Delić T Moškrič A Fišer C (2015) Molecular phylogeny of the subterranean genus *Niphargus* (Crustacea: Amphipoda) in the Middle East: a comparison with European Niphargids. Zoological Journal of the Linnean Society 175(4): 812–826. 10.1111/zoj.12296

[B18] Federov PV (1971) Postglacial transgression of the Black Sea. International Geology Review 14(2): 160–164. 10.1080/00206817209475678

[B19] Fišer C (2012) *Niphargus*: A model system for evolution and ecology. In: Culver DC White WB (Eds) Encyclopedia of Caves.Academic Press, New York, 555–564. 10.1016/B978-0-12-383832-2.00082-7

[B20] Fišer C Sket B Stoch F (2006) Distribution of four narrowly endemic *Niphargus* species (Crustacea: Amphipoda) in the western Dinaric region with description of a new species. Zoologischer Anzeiger 245(2): 77–94. 10.1016/j.jcz.2006.05.003

[B21] Fišer C Sket B Trontelj P (2008) A phylogenetic perspective on 160 years of troubled taxonomy of *Niphargus* (Crustacea: Amphipoda). Zoologica Scripta 37(6): 665e680. 10.1111/j.1463-6409.2008.00347.x

[B22] Fišer C Pipan T Culver DC (2014) The vertical extent of groundwater metazoans: An ecological and evolutionary perspective. Bioscience 64(11): 971–979. 10.1093/biosci/biu148

[B23] Fišer C Robinson CT Malard F (2018) Cryptic species as a window into the paradigm shift of the species concept. Molecular Ecology 27(3): 613–635. 10.1111/mec.14486 29334414

[B24] Fišer C Delić T Luštrik R Zagmajster M Altermatt F (2019) Niches within a niche: Ecological differentiation of subterranean amphipods across Europe’s interstitial waters. Ecography 42(6): 1212–1223. 10.1111/ecog.03983

[B25] Folmer O Black M Hoeh W Lutz R Vrijenhoek R (1993) DNA primers for amplification of mitochondrial cytochrome c oxidase subunit I from diverse metazoan invertebrates. Molecular Marine Biology and Biotechnology 3(5): 294–299. 10.1371/journal.pone.0013102 7881515

[B26] Foulquier A Malard F Lefébure T Douady ChJ Gibert J (2008) The Imprint of Quaternary Glaciers on the Present-Day Distribution of the Obligate Groundwater Amphipod *Niphargus virei* (Niphargidae). Journal of Biogeography 35(3): 552–564. 10.1111/j.1365-2699.2007.01795.x

[B27] Georgievski G Stanev EV (2006) Paleo-evolution of the Black Sea watershed: Sea level and water transport through the Bosporus Straits as an indicator of the Late Glacial-Holocene transition. Climate Dynamics 26(6): 631–644. 10.1007/s00382-006-0123-y

[B28] Guindon S Dufayard JF Lefort V Anisimova M Hordijk W Gascuel O (2010) New algorithms and methods to Estimate Maximum–Likelihood Phylogenies: Assessing the Performance of PhyML 3.0. Systematic Biology 59(3): 307–321. 10.1093/sysbio/syq010 20525638

[B29] Guy-Haim T Simon-Blecher N Frumkin A Naaman I Achituv Y (2018) Multiple transgressions and slow evolution shape the phylogeographic pattern of the blind cave-dwelling shrimp *Typhlocaris*. PeerJ 6: e5268. 10.7717/peerj.5268 PMC6061184 30057861

[B30] Hebert PD Cywinska A Ball SL de Waard JR (2003) Biological identifications through DNA barcodes. Proceedings. Biological Sciences 270(1512): 313–321. 10.1098/rspb.2002.2218 12614582 PMC1691236

[B31] Hewitt GM (2000) The genetic legacy of the Quaternary ice ages. Nature 405: 907e913. 10.1038/35016000 10879524

[B32] Horton T Lowry J De Broyer C Bellan-Santini D Copilas-Ciocianu D Corbari L Costello MJ Daneliya M Dauvin J-C Fišer C Gasca R Grabowski M Guerra-García JM Hendrycks E Hughes L Jaume D Jazdzewski K Kim Y-H King R Krapp-Schickel T LeCroy S Lörz A-N Mamos T Senna AR Serejo C Souza-Filho JF Tandberg AH Thomas JD Thurston M Vader W Väinölä R Valls Domedel G Vonk R White K Zeidler W (2024) World Amphipoda Database. *Niphargus* Schiödte, 1849. World Register of Marine Species at: https://www.marinespecies.org/aphia.php?p=taxdetails&id=545672 [accessed 1 Dec 2024]

[B33] Jablonski D Kukushkin OV Avci A Bunyatova S Kumlutaş Y Ilgaz C Polyakova E Shiryaev K Tuniyev B Jandzik D (2019) The biogeography of *Elaphe sauromates* (Pallas, 1814), with a description of a new rat snake species. PeerJ 7: e6944. 10.7717/peerj.6944 PMC6544014 31179175

[B34] Karaman GS (1986) Redescription of subterranean gammaridean species *Niphargus longicaudatus* (Costa 1851) based on topotypic material (Contribution to the Knowledge of the Amphipoda 161). Fragmenta Balcanica 13: 27–42.

[B35] Karaman GS (1989) New species of the family Niphargidae and new localities of some other subterranean Gammaridean species from Yugoslavia (Contribution to the knowledge of the Amphipoda 166). Glasnik republičkog zavoda za zaštitu prirode I prirodnjačkog muzeja u Titogradu 19: 15–32.

[B36] Karaman GS (2012) 256. Contribution to knowledge of Amphipoda. New species of the subterranean genus *Niphargus* Schiödte, 1849 (Amphipoda, Gammaridea, Niphargidae) from Russia, *N. krasnodarus* sp. n. Biologia Serbica 34(1): 12–16.

[B37] Keppel G Van Niel KP Wardell-Johnson GW Yates CJ Byrne M Mucina L Schut AGT Hopper SD Franklin SE (2012) Refugia: identifying and understanding safe havens for biodiversity under climate change. Global Ecology and Biogeography 21: 393e404. 10.1111/j.1466-8238.2011.00686.x

[B38] Kimura M (1980) A simple method for estimating evolutionary rates of base substitutions through comparative studies of nucleotide sequences. Journal of Molecular Evolution 16(2): 111–120. 10.1007/BF01731581 7463489

[B39] Kokalj AJ Fišer Ž Dolar A Novak S Drobne D Bračko G Fišer C (2022) Screening of NaCl salinity sensitivity across eight species of subterranean amphipod genus *Niphargus*. Ecotoxicology and Environmental Safety 236: e113456. 10.1016/j.ecoenv.2022.113456 35395599

[B40] Krever V Zazanashvili N Jungius H Williams L Petelin D (2001) Biodiversity of the Caucasus Ecoregion. World Wide Fund for Nature, Moscow, 132 pp.

[B41] Kumar S Stecher G Tamura K (2016) MEGA 7: Molecular Evolutionary Genetics Analysis version 7.0 for bigger datasets. Molecular Biology and Evolution 33(7): 1870–1874. 10.1093/molbev/msw054 27004904 PMC8210823

[B42] Lefébure T Douady ChJ Gouy M Trontelj P Briolay J Gibert J (2006) Phylogeography of a subterranean amphipod reveals cryptic diversity and dynamic evolution in extreme environments. Molecular Ecology 15(7): 1797–1806. 10.1111/j.1365-294X.2006.02888.x 16689899

[B43] Lefébure T Douady ChJ Malard F Gibert J (2007) Testing dispersal and cryptic diversity in a widely distributed groundwater amphipod (*Niphargus rhenorhodanensis*). Molecular Phylogenetics and Evolution 42(3): 676–686. 10.1016/j.ympev.2006.08.020 17049283

[B44] Leigh JW Bryant D (2015) PopART: Full-feature software for haplotype network construction. Methods in Ecology and Evolution 6(9): 1110–1116. 10.1111/2041-210X.12410

[B45] Marin IN (2017) COXI based phylogenetic analysis of Caucasian clade of European *Troglocaris* s.l. (Crustacea: Decapoda: Atyidae) with the suggestion of a new taxonomic group structure. Biosystems Diversity 25(4): 323–327. 10.15421/011749

[B46] Marin IN (2019) Crustacean “cave fishes” from the Arabika karst massif (Abkhazia, Western Caucasus): New species of stygobiotic crustacean genera *Xiphocaridinella* and *Niphargus* from the Gegskaya Cave and adjacent area. Arthropoda Selecta 28(2): 225–245. 10.15298/arthsel.28.2.05

[B47] Marin IN Palatov DM (2022) *Dursogammarus dromaderus* gen. et sp. nov., a new Ponto-Caspian gammarid (Amphipoda: Gammaridae) from the coastal pebble habitats of the foothills of the Caucasus. Zoology in the Middle East 68(3): 237–246. 10.1080/09397140.2022.2116171

[B48] Marin IN Palatov DM (2023) Insights on the Existence of Ancient Glacial Refugee in the Northern Black/Azov Sea Lowland, with the Description of the First Stygobiotic Microcrustacean Species of the Genus *Niphargus* Schiödte, 1849 from the Mouth of the Don River. Diversity 15(5): e682. 10.3390/d15050682

[B49] Marin IN Palatov DM (2024) The Diversity of Freshwater Stygobiotic Crustaceans in the Republic of North Ossetia–Alania Provides New Evidence for the Existence of an Ancient Glacial Refugium in the North Caucasus Region. Water 16(9): e1212. 10.3390/w16091212

[B50] Marin IN Palatov DM (2025) Stygobiotic species of the genus *Niphargus* Schiödte, 1849 (Amphipoda: Niphargidae) from the Republic of Adygea, with a review and validation of the species from the northern slope of the Great Caucasian Ridge. Arthropoda Selecta 34(2): 241–257. 10.15298/arthsel.34.2.08

[B51] Marin I Krylenko S Palatov D (2021a) Euxinian relict amphipods of the Eastern Paratethys in the subterranean fauna of coastal habitats of the Northern Black Sea region. Zoologia Bespozvonocnyh 18(3): 247–320. 10.15298/invertzool.18.3.05

[B52] Marin I Krylenko S Palatov D (2021b) The Caucasian relicts: a new species of the genus *Niphargus* (Crustacea: Amphipoda: Niphargidae) from the Gelendzhik–Tuapse area of the Russian southwestern Caucasus. Zootaxa 4963(3): 483–504. 10.11646/zootaxa.4963.3.5 33903542

[B53] Marin IN Turbanov I Prokopov G Palatov D (2022) A New Species of the Genus *Niphargus* Schiödte, 1849 (Crustacea: Amphipoda: Niphargidae) from Groundwater Habitats of the Tarkhankut Upland, Crimean Peninsula. Diversity 14(12): e1010. 10.3390/d14121010

[B54] Marin I Palatov D Copilaş-Ciocianu D (2023a) The remarkable Ponto-Caspian amphipod diversity of the lower Durso River (SW Caucasus) with the description of *Litorogammarus dursi* gen. et sp. nov. Zootaxa 5297(4): 483–517. 10.11646/zootaxa.5297.4.2 37518782

[B55] Marin IN Barjadze S Maghradze E Palatov DM (2023b) Diversity, taxonomy and phylogenetic relationships of the “*Niphargus borutzkyi*” ingroup (Crustacea: Amphipoda: Niphargidae) in Western Georgia, SW Caucasus. Zootaxa 5352(4): 477–500. 10.11646/zootaxa.5352.4.2 38221433

[B56] McInerney CI Maurice L Robertson AL Knight LRFD Arnscheidt J Venditti C Dooley JSG Mathers T Matthijs S Eriksson K Proudlove GS Hänfling B (2014) The Ancient Britons: Groundwater fauna survived extreme climate changes over tens of millions of years across NW Europe. Molecular Ecology 23(5): 1153–1166. 10.1111/mec.12664 24433175

[B57] McKinney ML (1997) Extinction Vulnerability and Selectivity: Combining Ecological and Paleontological Views. Annual Review of Ecology and Systematics 28(1): 495–516. 10.1146/annurev.ecolsys.28.1.495

[B58] Müller P Geary DH Magyar I (1999) The endemic molluscs of the Late Miocene Lake Pannon: Their origin, evolution and family-level taxonomy. Lethaia 32(1): 47–60. 10.1111/j.1502-3931.1999.tb00580.x

[B59] Myers N Mittermeier RA Mittermeier CG da Fonseca GAB Kent J (2000) Biodiversity hotspots for conservation priorities. Nature 403(6772): 853–858. 10.1038/35002501 10706275

[B60] Neubauer TA Harzhauser M Kroh A Georgopoulou E Mandic O (2015) A gastropod-based biogeographic scheme for the European Neogene freshwater systems. Earth-Science Reviews 143(29): 98–116. 10.1016/j.earscirev.2015.01.010

[B61] Palatov D Marin N (2021) Epigean (pond-dwelling) species of the genus *Niphargus* Schiödte, 1849 (Crustacea, Amphipoda, Niphargidae) from the coastal plains of the Black and Azov seas of the north- and south-western Caucasus. Zoologia Bespozvonocnyh 18(2): 105–151. 10.15298/invertzool.18.2.05

[B62] Parvizi E Keikhosravi A Solhjouy-Fard S Sheybak F Schubart Ch (2019) Phylogeography of *Potamon ibericum* (Brachyura: Potamidae) identifies Quaternary glacial refugia within the Caucasus biodiversity hot spot. Ecology and Evolution 9(1): 1–11. 10.1002/ece3.5078 PMC6476761 31031941

[B63] Popov SV Rögl F Rozanov AY Steininger FF Shcherba IG Kovac M (2004) Lithological–Paleogeographic maps of the Paratethys. 10 maps Late Eocene to Pliocene. Courier Forschungsinstitut Senckenberg 250: 1–46.

[B64] Popov SV Shcherba IG Ilyina LB Nevesskaya LA Paramonova NP Khondkarian SO Magyar I (2006) Late Miocene to Pliocene palaeogeography of the Paratethys and its relation to the Mediterranean. Palaeogeography, Palaeoclimatology, Palaeoecology 238(1–4): 91–106. 10.1016/j.palaeo.2006.03.020

[B65] Rebijith KB Asokan R Kumar NK Krishna V Chaitanya N Ramamurthy VV (2013) DNA barcoding and elucidation of cryptic aphid species (Hemiptera: Aphididae) in India. Bulletin of Entomological Research 103(5): 601–610. 10.1017/S0007485313000278 23680306

[B66] Ryan WBF Pitman WCIII III Major CO Shimkus K Moskalenko V Jones GA Dimitrov P Gorür N Sakinç M Yüce H (1997) An abrupt drowning of the Black Sea shelf. Marine Geology 138(1–2): 119–126. 10.1016/S0025-3227(97)00007-8

[B67] Sars GO (1894) . Crustacea caspia. Contributions to the knowledge of the Carcinological Fauna of the Caspian Sea. Part III. Amphipoda. Gammarida. Bulletin de l’Academie Imperiale des Sciences de St.-Petersbourg (Ser. 5) 1: 179–223. [343–378.] 10.5962/bhl.title.10631

[B68] Schmitt T (2007) Molecular biogeography of Europe: Pleistocene cycles and postglacial trends. Frontiers in Zoology 4 (11). 10.1186/1742-9994-4-11 PMC1868914 17439649

[B69] Shatilova I Mchedlishvili N Rukhadze L Kvavadze E (2011) The history of the flora and vegetation of Georgia (South Caucasus). Georgian National Museum, Institute of Paleobiology, Tbilisi, 200 pp.

[B70] Sket B (1977) *Niphargus* im brackwasser. Studies on Gammaridea. Proceedings of the Third International Colloquium on *Gammarus* and *Niphargus*, Schlitz, 1975. Crustaceana, Supplements 4: 188–191. 10.1163/9789004629332_021

[B71] Tomilova AA Lyubas AA Kondakov AV Vikhrev IV Gofarov MY Kolosova YS Vinarski MV Palatov DM Bolotov IN (2020) Evidence for Plio-Pleistocene Duck Mussel Refugia in the Azov Sea River Basins. Diversity 12(3): e118. 10.3390/d12030118

[B72] Trontelj P Douady ChJ Fišer C Gibert J Gorički Š Lefébure T Sket B Zakšek V (2009) A molecular test for cryptic diversity in ground water: How large are the ranges of macro-stygobionts? Freshwater Biology 54(4): 727–744. 10.1111/j.1365-2427.2007.01877.x

[B73] Väinölä R Witt JDS Grabowski M Bradbury JH Jazdzewski K Sket B (2008) Global diversity of amphipods (Amphipoda; Crustacea) in freshwater. Hydrobiologia 595(1): 241–255. 10.1007/s10750-007-9020-6

[B74] van Leeuwen CHA Huig N van der Velde G van Alen TA Wagemaker CAM Sherman CDH Klaassen M Figuerola J (2013) How did this snail get here? Several dispersal vectors inferred for an aquatic invasive species. Freshwater Biology 58(1): 88–99. 10.1111/fwb.12041

